# Terahertz Spectra of Mannitol and Erythritol: A Joint Experimental and Computational Study

**DOI:** 10.3390/molecules29133154

**Published:** 2024-07-02

**Authors:** Zeyu Hou, Bingxin Yan, Yuhan Zhao, Bo Peng, Shengbo Zhang, Bo Su, Kai Li, Cunlin Zhang

**Affiliations:** 1 Department of Physics, Capital Normal University, Beijing 100048, China; 18135927761@163.com (Z.H.); bingxiny2022@163.com (B.Y.); 2220602038@cnu.edu.cn (Y.Z.); 2210602051@cnu.edu.cn (B.P.); zhangshengbo@cnu.edu.cn (S.Z.); zclcnu@163.com (C.Z.); 2 Beijing Key Laboratory for Terahertz Spectroscopy and Imaging, Beijing 100048, China; 3 Key Laboratory of Terahertz Optoelectronics, Ministry of Education, Beijing 100048, China; 4 Department of Chemistry, Capital Normal University, Beijing 100048, China; likai123452024@163.com

**Keywords:** THz time-domain spectroscopy, density functional theory, microfluidic chip, PXRD, Raman

## Abstract

Sugar substitutes, which generally refer to a class of food additives, mostly have vibration frequencies within the terahertz (THz) band. Therefore, THz technology can be used to analyze their molecular properties. To understand the characteristics of sugar substitutes, this study selected mannitol and erythritol as representatives. Firstly, PXRD and Raman techniques were used to determine the crystal structure and purity of mannitol and erythritol. Then, the THz time-domain spectroscopy (THz-TDS) system was employed to measure the spectral properties of the two sugar substitutes. Additionally, density functional theory (DFT) was utilized to simulate the crystal configurations of mannitol and erythritol. The experimental results showed good agreement with the simulation results. Finally, microfluidic chip technology was used to measure the THz spectroscopic properties of the two sugar substitutes in solution. A comparison was made between their solid state and aqueous solution state, revealing a strong correlation between the THz spectra of the two sugar substitutes in both states. Additionally, it was found that the THz spectrum of a substance in solution is related to its concentration. This study provides a reference for the analysis of sugar substitutes.

## 1. Introduction

Powder X-ray Diffraction (PXRD) is an efficient, accurate, and non-destructive quantitative method for determining crystal forms [1], making it suitable for areas such as pharmaceutical quality control and crystal structure research [2]. It can determine the crystal form and structure of drugs by providing unit cell parameters such as interatomic distances and bond angles [3], which aids in the qualitative analysis of distinguishing between crystalline and amorphous states, as well as mixtures and compounds. Raman spectroscopy is a type of scattering spectroscopy [4]. It is an analytical method based on the Raman scattering effect, which involves the analysis of scattered light at frequencies different from the incident light to obtain information about molecular vibrations and rotations [5]. This technique is applied in the study of molecular structures. Raman spectroscopy has the advantages of being fast, accurate, non-destructive, and applicable to various samples, making it widely used in fields such as chemical analysis, materials science, and biology [6]. THz radiation, also known as far-infrared radiation, is a type of high-frequency electromagnetic wave. Its frequency typically falls within the range of 0.1–10 THz, corresponding to a wavelength range of 3000–30 μm. Due to the rapid development of ultrafast laser technology and semiconductor material technology in recent years, reliable and stable excitation light sources have been produced, enabling THz waves to be widely used in the fields of security detection, biomedicine, and spectroscopy [7]. The photon energy of THz waves is relatively low and cannot ionize samples, making them suitable for non-destructive testing of materials [8]. The use of THz waves for material inspection offers the advantages of safety and reliability [9]. THz spectroscopy has been widely applied in structural identification due to its high sensitivity not only in discriminating molecules themselves but also in detecting any changes in their structure. Most organic molecules, including sugar substitute molecules [10], exhibit rich and unique spectra in the frequency region below 4 THz [11]. Therefore, combining these three technologies for material detection will yield more accurate and reasonable results. 

Mannitol is an excellent functional sugar alcohol. It has a sweet taste, with a sweetness intensity that is 0.55–0.65 times that of sucrose [12]. Among functional sugar alcohols, mannitol is the only one that is not easily hygroscopic and is a six-carbon sugar alcohol. Erythritol is a new type of polyol sweetener with a taste similar to sucrose. Its relative sweetness is 70–80% of that of sucrose. Compared with other sugar substitutes, these two types of sugar have the advantages of good stability, low calorie content, high safety factor, and suitable price [13]. They are typical representatives of sugar substitutes used in daily life and have wide commercial applications. For example, erythritol and mannitol are widely used as food additives in the fields of table sugar, flavoring sugar, beverages, candy foods, and baked goods. They account for over 70% of these products [14]. At the same time, compared to carbohydrates, these two types of sugar substitutes have advantages such as low energy, good sweetness, good taste, antioxidant properties, and improved metabolism. They are also beneficial to health and have a wide range of applications in the pharmaceutical field [15]. Erythritol is an ideal sweetener for patients with diabetes, and mannitol is one of the clinical rescue drugs, so these two sugar substitutes have high research value.

There are significant differences in the application of the two types of sugar substitutes in solid and solution states. Firstly, in terms of processing and storage, solid sugar substitutes can be easier to control during processing and storage due to their solid-state characteristics, reducing the risk of contamination and spoilage [16]. Liquid sugar substitutes require stricter storage conditions to prevent microbial contamination and spoilage. Secondly, in terms of health, solid sugar substitutes are not easy to absorb and digest, which can cause gastrointestinal discomfort. However, liquid sugar substitutes have extremely low calories and are easy to absorb, so they are more commonly used in daily life [17]. Therefore, we can understand the necessity of distinguishing and using solid and liquid sugar substitutes. When studying liquid samples, there are often accompanying issues such as high sample consumption and environmental pollution. To address these challenges, we have introduced microfluidic technology [18]. Microfluidic technology is a scientific technique characterized by its ability to precisely manipulate fluids at the microscale. Currently, microfluidic technology has been widely applied in fields such as chemistry, physics, and biological detection due to its advantages of low liquid sample consumption, fast detection speed, and easy operation [19]. In this study, PXRD technology, Raman spectroscopy, the THz-TDS system, and density functional theory (DFT) were employed to detect and analyze the vibrational absorption of mannitol and erythritol. Through experimental measurements of the vibrational absorption of these two sugar substitutes, it was found that they exhibit different THz absorption peaks, which are generally consistent with the anomalous dispersion observed in the refractive index spectra. And solid-state density functional theory (DFT) software (Materials Studio 2019) was utilized to simulate their intermolecular vibrational modes. In addition, microfluidic chip technology was employed to measure the spectral characteristics of the two sugar substitutes in solution, and a comparison was made between their solid and liquid states. It was found that there is a correlation between the same substance in different states, while there are differences in the THz absorption peaks of the two substances in solution. Finally, we compared the spectra of different concentrations and found that the THz absorption spectra of the same substance in solution are correlated with its concentration. These results, using mannitol and erythritol as examples, provide their spectral characteristics in the low-frequency THz range and lay a foundation for the application of THz technology in the study of food additives. 

## 2. Results and Discussion

### 2.1. PXRD Characterization and Analysis of Two Sugar Substitutes

**Figure 1:**
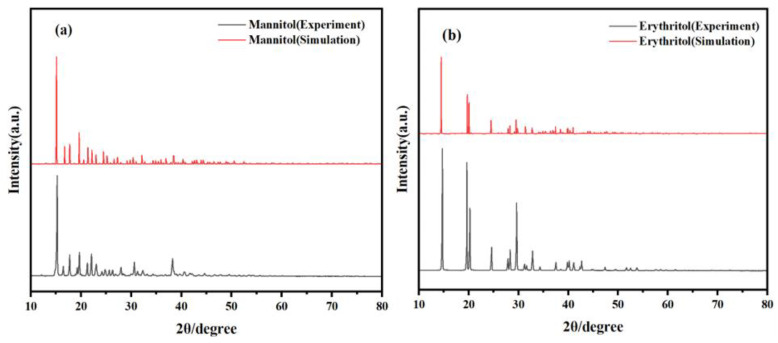
Comparison of PXRD spectra between experimental results and theoretical calculations. (**a**) Mannitol and (**b**) erythritol.

PXRD is an extremely useful technique for studying the crystal structure of materials. It offers several advantages, including the ability to determine crystal structures, rapid and non-destructive analysis, quantitative evaluations, and applicability to polycrystalline materials. To ensure the validity and reasonableness of the crystal cells for the two sugar alcohols, mannitol and erythritol, in computational simulations, the fundamental structural information of these compounds was first verified using X-ray diffraction spectroscopy. As shown in Figure 1, the X-ray diffraction peaks for mannitol are observed at 15.25°, 17.74°, 19.55°, 21.21°, 22.14°, 22.90°, 30.51°, and 38.27°. The X-ray diffraction peaks for erythritol are observed at 14.63°, 19.63°, 20.21°, 24.54°, 28.35°, 29.58°, and 32.86°. At the same time, through comparison, the simulated PXRD spectra match very well with the experimental spectra of mannitol and erythritol. This strong agreement validates the structural models used in the computational simulations and confirms their accuracy in representing the real-world crystal structures of these sugar alcohols. 

### 2.2. Raman Spectroscopic Characterization and Analysis of Two Sugar Substitutes

**Figure 2:**
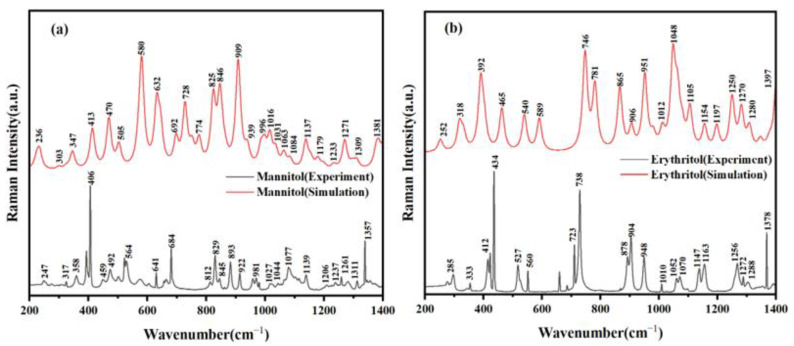
Comparison of Raman spectra between experimental results and theoretical calculations. (**a**) Mannitol and (**b**) erythritol.

The analytical method of Raman spectroscopy does not require the pretreatment or preparation of the sample, avoiding the introduction of errors. It achieves non-destructive qualitative and quantitative analysis. Moreover, Raman spectroscopy boasts advantages such as simple operation, short measurement times, and high sensitivity during the analytical process [20]. Through the analysis of mannitol and erythritol using Raman spectroscopy, more molecular-level information can be obtained [21]. Figure 2 shows the Raman spectra of mannitol and erythritol in the range of 200–1400 cm^−1^. It can be observed that mannitol and erythritol exhibit distinct absorption peaks in the frequency domain. Mannitol displays absorption peaks at 247, 317, 358, 406, 459, 492, 564, 641, 684, 812, 829, 845, 893, 922, 981, 1027, 1044, 1077, 1139, 1206, 1237, 1261, 1311, and 1357 cm^−1^. On the other hand, erythritol has absorption peaks at 285, 333, 412, 434, 527, 560, 723, 738, 878, 904, 948, 1010, 1052, 1070, 1147, 1163, 1256, 1272, 1288, and 1378 cm^−1^. At the same time, through comparison, the simulated Raman spectra agree well with the experimental spectra. This indicates that the samples used in the experiment have high purity and did not undergo any chemical reactions during the analysis. This provides assurance for further studies on these two sugar alcohols, mannitol, and erythritol, as accurate and reliable spectral data are crucial for understanding their properties and behaviors.

### 2.3. THz Spectra of Two Solid Sugar Substitutes

**Figure 3:**
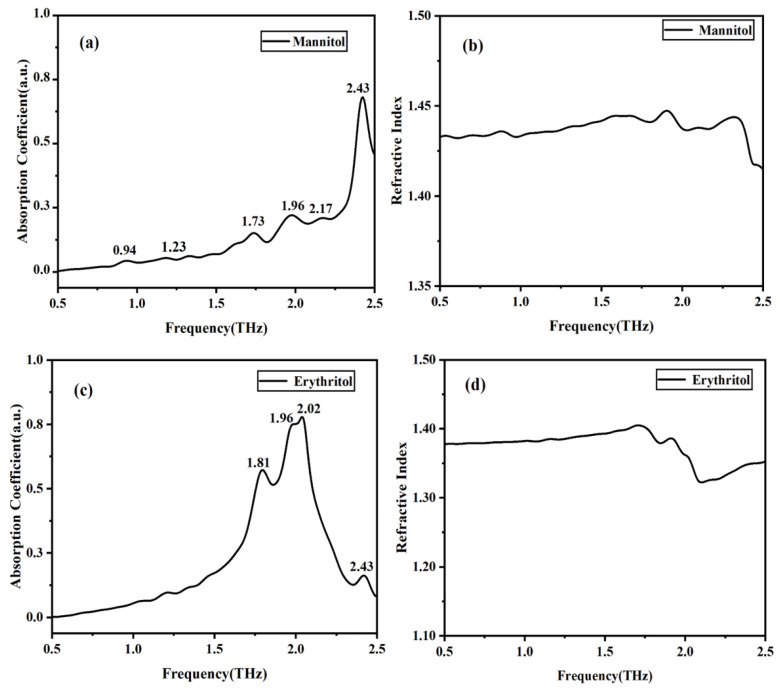
THz absorption and refractive index spectra of solid mannitol and erythritol. (**a**) THz absorption spectrum of mannitol. (**b**) Refractive index of mannitol. (**c**) THz absorption spectrum of erythritol. (**d**) Refractive index of erythritol.

The absorption coefficient and refractive index spectra can be obtained by performing fast Fourier transform on the time-domain spectra of the sample and reference signals. Figure 3 shows the THz spectra of solid mannitol and erythritol at room temperature. It can be observed that mannitol has five distinct characteristic absorption peaks in the THz frequency range, located at 0.94, 1.73, 1.96, 2.17, and 2.43 THz, respectively. Due to the different mechanisms of peak formation, the intensities of these peaks also vary. Erythritol exhibits four absorption peaks at 1.81, 1.96, 2.02, and 2.43 THz. The refractive index of mannitol ranges between 1.40 and 1.45, while that of erythritol falls between 1.30 and 1.40. At the positions of the absorption peaks, the refractive index decreases with increasing frequency, indicating anomalous dispersion. This behavior is consistent with the Kramers–Kronig relations. 

### 2.4. Simulation of Two Solid Sugar Substitutes

**Figure 4:**
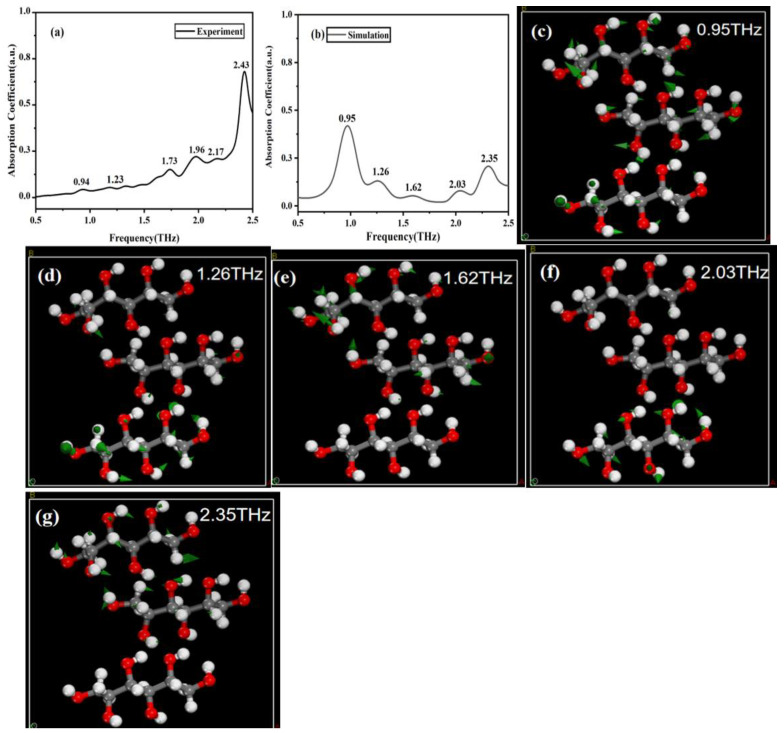
Comparison of THz spectroscopy and corresponding molecular vibration modes. (**a**) Experimental spectrum of mannitol. (**b**) Simulation spectrum of mannitol. (**c**–**g**) Vibration modes of mannitol at 0.95, 1.26, 1.62, 2.03, and 2.35 THz, respectively.

In order to better understand the spectral mechanisms of mannitol and erythritol, as well as the relationship between THz spectra and molecular vibrations, we conducted simulation calculations for these two sugar substitutes. Figure 4a,b show the experimental and simulated THz spectra of mannitol, respectively. Through simulation calculations, we found that the simulated absorption peaks of solid mannitol are located at 0.95, 1.26, 1.62, 2.03, and 2.35 THz, which are basically consistent with the experimental peaks. The vibration modes of this structure are shown in Figure 4c–g. Calculations indicate that the absorption peaks are attributed to the vibrations of functional groups such as -CH_2_OH and -OH as well as collective molecular vibrations or intermolecular interactions. It is worth noting that intermolecular interactions, particularly hydrogen bonding, can influence the absorption peaks of THz radiation. Therefore, the simulated absorption peaks may slightly differ from those observed experimentally. Additionally, in the calculations, the peaks at 1.96 and 2.17 THz were merged into a single peak at 2.03 THz. The reason for this situation may be that during the experimental process, due to various factors such as equipment accuracy, environmental interference, and operational errors, there may be certain errors in the experimental results, which is difficult to avoid. However, our simulation was conducted under absolute conditions, and the control of parameter settings and calculation methods is more precise, which may avoid certain experimental errors. Therefore, even if the two should theoretically obtain similar results, the actual curves obtained may exhibit different widths due to the existence of experimental errors. Meanwhile, due to the presence of errors, the two peak frequencies may overlap when they are relatively close.

**Figure 5:**
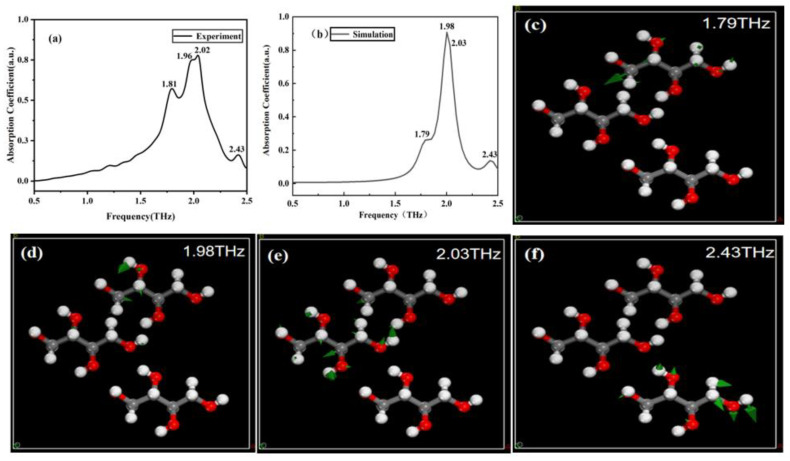
Comparison of THz spectroscopy and corresponding molecular vibration modes. (**a**) Experimental spectrum of erythritol. (**b**) Simulation spectrum of erythritol. (**c**–**f**) Vibration modes of erythritol at 1.79, 1.98, 2.03, and 2.43 THz, respectively.

Figure 5a,b depict the experimental and simulated THz spectra of erythritol, respectively. Through simulation calculations, we identified the simulated absorption peaks of erythritol at 1.79, 1.98, 2.03, and 2.43 THz. These four peaks closely correspond to the experimental results, where the simulated peaks at 1.79, 1.98, and 2.03 THz align with the experimental peaks at 1.81, 1.96, and 2.02 THz, respectively. The simulated peak at 2.43 THz directly corresponds to an experimental peak too. Therefore, it is initially determined that these four peaks are the characteristic peaks of erythritol, and their vibration modes are shown in Figure 5c–f, respectively. 

The differences between experimental and simulation results can be explained by several factors. Firstly, the differences can be attributed to the fact that we adopted an ideal crystal structure in our calculations, which is difficult to achieve in actual experimental measurements. Secondly, the absorption peaks of THz radiation can be influenced by intermolecular interactions, especially the effects of hydrogen bonding. These molecular interactions may not be fully captured in simulation calculations, leading to slight variations in the absorption peaks. The detailed vibrational assignments of the two sugar substitutes in the THz frequency range are provided in Table 1.

### 2.5. THz Spectra of Two Sugar Substitute Solutions

**Figure 6:**
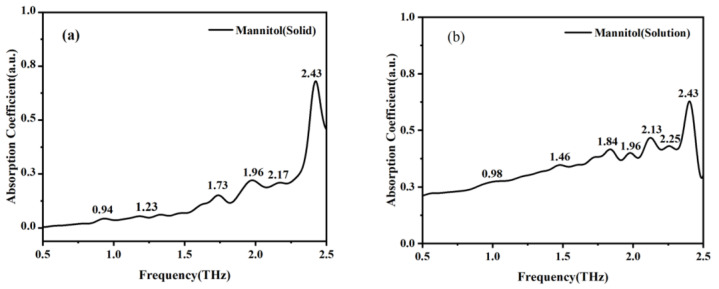
Absorption coefficient spectra of solid mannitol (**a**) and mannitol in solution state (**b**).

**Figure 7:**
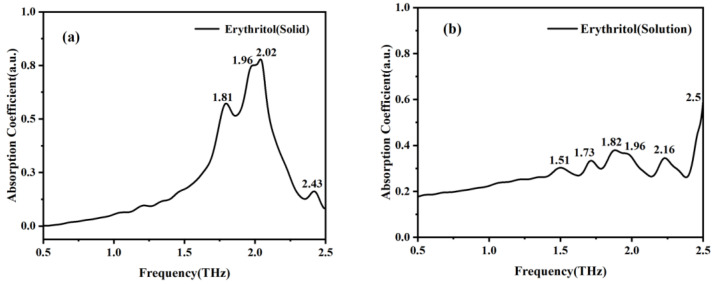
Absorption coefficient spectra of solid erythritol (**a**) and erythritol in solution state (**b**).

As most sugar substitutes function in solution, in order to better study the characteristics of mannitol and erythritol in solution, we used the THz-TDS system and microfluidic chip to measure these two samples. The absorption peaks of solid mannitol at 0.94, 1.23, 1.73, 1.96, 2.17, and 2.43 THz in Figure 6a correspond to the absorption peaks of mannitol solution at 0.98, 1.46, 1.84, 1.96, 2.13, and 2.43 THz in Figure 6b. Similarly, the absorption peaks of solid erythritol at 1.81, 1.96, 2.02, and 2.43 THz shown in Figure 7a are correlated with the absorption peaks of erythritol in solution at 1.82, 1.96, 2.16, and 2.5 THz in Figure 7b. The absorption peaks of the two sugar substitutes in solid and solution states are basically consistent. These findings indicate a strong correlation between the spectral characteristics of mannitol in solid and solution states. However, the absorption peak of mannitol solution at 2.25 THz is not present in the THz absorption spectrum of solid mannitol, and the absorption peaks of erythritol solution at 1.51 and 1.73 THz are absent in the THz absorption spectrum of solid erythritol. This is because in solid matter, the interactions and arrangement between molecules are relatively stable, while in solution, the molecules are in a liquid environment, and the intermolecular interactions and arrangement are relatively complex and dynamic, resulting in more absorption peaks in the THz band of the solution. Meanwhile, in solution, there may be interactions such as hydrogen bonding and van der Waals forces between solute molecules and water molecules, as well as between solute molecules [22]. These interactions will affect the vibrational and rotational modes of molecules, resulting in more absorption peaks in the THz band. The reason why erythritol undergoes greater changes than mannitol in both solid and solution states is that its solubility is greater than that of mannitol at room temperature [23]. This indicates that the molecular difference between erythritol in solid and solution states is greater than that between mannitol in solid and solution states. These differences may result in different vibrational and rotational modes of molecules in the THz band, resulting in different absorption peaks [24].

**Figure 8:**
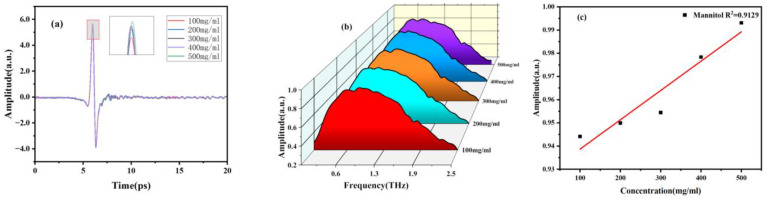
THz spectra of different concentrations of mannitol solutions. (**a**) Time-domain spectra. (**b**) Frequency-domain spectra. (**c**) Signal strength concentration relationship diagram.

**Figure 9:**
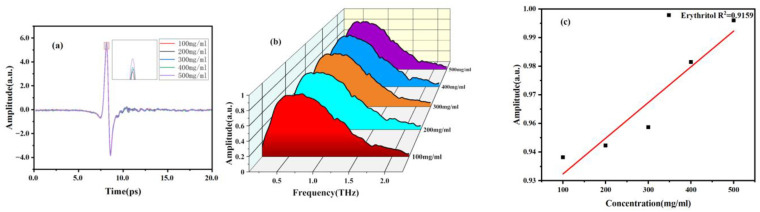
THz spectra of different concentrations of erythritol solutions. (**a**) Time-domain spectra. (**b**) Frequency-domain spectra. (**c**) Signal strength concentration relationship diagram.

In addition, we also investigated the correlation between concentration and spectrum. Firstly, sample solutions with different concentration gradients were prepared and injected into the microfluidic chip. The THz spectra of mannitol and erythritol solutions at different concentrations were measured at room temperature. The THz time-domain and frequency-domain spectra are shown in Figure 8 and Figure 9. It can be observed that as the concentration of mannitol and erythritol increases, both the time-domain and frequency-domain spectra show relative enhancement, indicating reduced absorption and increased transmittance. The reason for this phenomenon is that as the concentration of the two sugar substitutes increases, the molecular content of mannitol and erythritol increases while the water molecule content decreases, resulting in reduced water absorption. Clearly, the THz spectra of the two sugar substitute solutions are influenced by the molecular content of mannitol or erythritol. It should be noted that that water molecules are present in liquid samples, and the high absorption of water molecules for THz will greatly affect the absorption peaks of the samples. Therefore, this experiment did not compare the absorption coefficients at different concentrations. 

## 3. Experiment

### 3.1. THz Time-Domain Spectroscopy System

**Figure 10:**
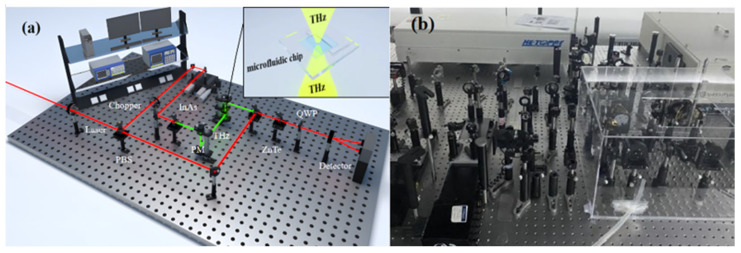
THz time-domain spectroscopy system. (**a**) Schematic diagram and (**b**) physical diagram.

The THz time-domain spectroscopy system used in the experiment is composed of a femtosecond laser, a THz radiation generation device, a THz wave detection device, and a time-delay control system, as shown in Figure 10. In the figure, the red line represents the laser, and the light green line represents THz. The femtosecond laser in this system generates a laser beam with a wavelength of 800 nm, a pulse width of 75 fs, and a repetition rate of 100 MHz. To ensure the accuracy of the experiment, the entire procedure was conducted in a nitrogen-filled environment at room temperature, with a relative humidity of 19.8%. During the experiment, the sample was fixed between off-axis parabolic mirrors. The laser beam, after passing through a half-wave plate (HWP) and a polarizing beam splitter (PBS), is divided into a pump pulse and a probe pulse. The pump pulse passes through a ZnTe crystal to generate THz waves, which then transmit through the sample and carry its information. Coherent detection is achieved by controlling the delay between the pump and probe pulses. The resulting optical signal is converted into an electrical signal, which is then amplified by a lock-in amplifier. Finally, the data from the entire experiment is collected and processed by a computer. 

### 3.2. Fabrication of Microfluidic Chip

**Figure 11:**
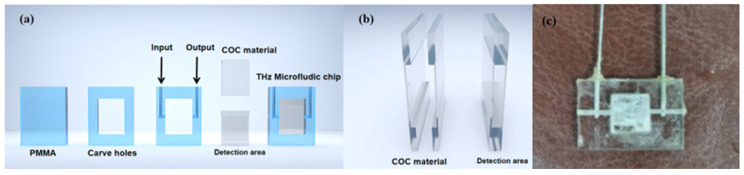
Microfluidic chip diagram. (**a**) Microfluidic chip preparation process. (**b**) Schematic diagram of THz detection area composed of COC. (**c**) Physical diagram of microfluidic chip.

The microfluidic chip used in the experiment is shown in Figure 11. Due to the high cost and scarcity of sheet-like COC (cyclic olefin copolymer) material, we only used COC in the detection area of the chip, while the remaining parts of the chip were fabricated using acrylic (PMMA). First, the cutting function of a laser engraving machine was used to cut polymethyl methacrylate into sizes of 40 × 40 × 2 mm^3^ thin film. Within this slice, a square hole of 20 × 20 × 2 mm^3^ was cut out to serve as the sample detection area. Next, the laser engraving machine’s dot-matrix function was used to create two cylindrical channels on the upper side of the PMMA, one on each side. These channels were 20 mm long and had a diameter of 0.6 mm. Additionally, two shorter channels, each 10 mm long and with the same diameter, were created on the left and right sides of the chip, perpendicular to the longer channels. Plasticine was then used to block off the ends of the shorter channels, forming two symmetrical L-shaped channels. The left channel served as the input, while the right channel functioned as the output [25]. To fabricate the sample cell, two pieces of COC material were prepared, each with dimensions of 20 × 20 mm and a uniform thickness of 1 mm. Using a high-precision milling machine, a 20 × 15 mm detection area was milled out in the center of both COC pieces. It is crucial to note that the milling process was performed on both pieces of COC material. The desired thickness of the detection area, which was the combined thickness of the two COC sample cells, was set to 50 μm. Therefore, the milling depth on each individual COC piece was precisely calculated to be 25 μm. Subsequently, the two COCs were bonded with acrylic adhesive and installed in the holes of PMMA. They were then sealed around with hot melt adhesive, as shown in Figure 11b. After completing the fabrication of the microfluidic device, it was necessary to test its sealing performance by pumping deionized water into the system.

### 3.3. Sample Preparation

**Figure 12:**
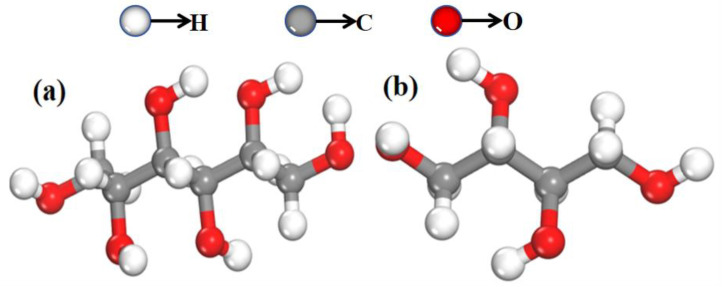
Molecular structure diagrams of two samples. (**a**) Mannitol and (**b**) erythritol.

The samples of mannitol and erythritol used in this study were purchased from Xiya Reagent Company (Chengdu, China) with a purity of 99.9% for both compounds. All samples were used as received without any further purification or adulteration. Polyethylene, provided by Sigma-Aldrich Trading Ltd. (Shanghai, China), is a white powder that exhibits excellent transmission of THz waves. It serves as an excellent reagent for testing and formulation purposes. After thorough grinding, 100 mg sample tablets with a diameter of 1 cm were prepared using mannitol, erythritol, and their mixture with polyethylene. These tablets were compressed under a pressure of 6.5 Mpa, resulting in tablets with thicknesses of 1.11 mm and 1.15 mm, respectively, for the different compositions. Subsequently, solutions of mannitol and erythritol were prepared using deionized water with a resistivity of 18.2 MΩ·cm. Within the concentration range of 0 to 1 g/mL, five different concentrations of both mannitol and erythritol solutions were prepared. The molecular structures of the two samples are shown in Figure 12a and Figure 12b, respectively. 

## 4. Analytical Methods and Theoretical Simulations

### 4.1. Data Processing

The data measured using the THz-TDS system constitute the time-domain signal of the sample. To obtain the corresponding frequency-domain signal, a Fourier transform is applied to the time-domain data. The formula for the Fourier transform from the time domain to the frequency domain is as follows:

(1) $$F\left(j\omega \right)=\int_{-\infty }^{+\infty }f\left(t\right)e^{-j\omega t}dt$$

where F(jω) is a frequency domain function, with *j* representing that this function is complex. f(t) is a time domain function and $e^{-j\omega t}$ is a frequency factor. The material absorption coefficient is calculated according to Lambert–Beer’s law. The sample’s absorption coefficient is calculated as follows:

(2) $$n_{s}\left(\omega \right)=1+\frac{c}{\omega d}\varphi \left(\omega \right)$$

(3) $$\alpha \left(\omega \right)=-\frac{2}{d}\ln\left\{\frac{\left|E_{echo0}\left(\omega \right)\right|}{\left|E_{ref}\left(\omega \right)\right|}\cdot \frac{{\left[n_{s}\left(\omega \right)+1\right]}^{2}}{4n_{s}\left(\omega \right)}\right\}$$

where *d* is the thickness of the experimental sample. $\left|E_{echo0}\left(\omega \right)\right|$ and $\left|E_{ref}\left(\omega \right)\right|$ are the Fourier transform amplitudes of the sample and reference signals, respectively. $n_{s}\left(\omega \right)$ is the sample’s refractive index, and φ(ω) is the phase difference of the Fourier transform between the sample and reference signal.

### 4.2. Simulation

**Figure 13:**
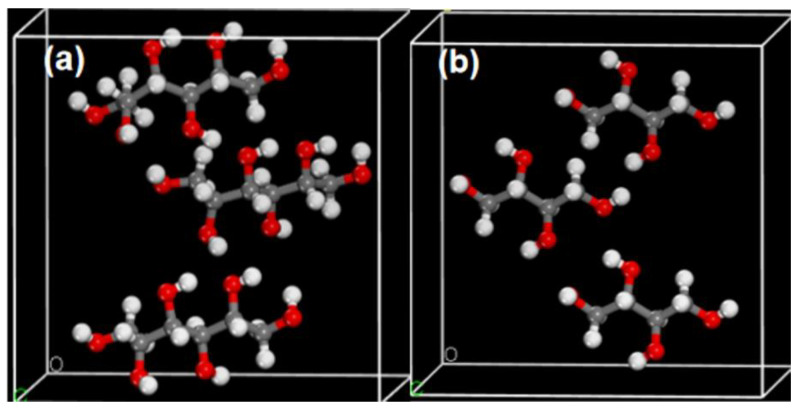
Crystal cell structure diagrams of mannitol (**a**) and erythritol (**b**).

To gain a deeper understanding of the spectroscopic mechanisms of mannitol and erythritol, as well as the relationship between THz spectra and molecular vibrations [26], we employed quantum chemical calculations based on density functional theory (DFT) to analyze and interpret the measured THz spectra [27]. All theoretical calculations were performed within the CASTEP module of Material Studio (MS) [28,29,30]. The specific settings for the kinetic energy cutoff in the simulation were as follows: Fine, 571.4 eV. The convergence criterion for geometric optimization was set to atom. The flag for sampling points K in the Brillouin zone was set to Fine 3 × 3 × 4. The simulated Task is Geometry optimization and Energy. The Relative treatment in the simulation is koelling-Harmon. At the same time, the Basis set is DNP+ and utilizing the Generalized Gradient Approximation (GGA) and Perdew–Burke–Ernzerhof (PBE) parameters for optimizing the molecular structures, energies, and frequencies of mannitol and erythritol. The unit cells for mannitol and erythritol are depicted in Figure 13.

## 5. Conclusions

In this study, PXRD technology, Raman technology, the THz-TDS system, and density functional theory (DFT) were used to detect and analyze the vibrational absorption of mannitol and erythritol. Through experimental measurements of the vibrational absorption of the two sugar substitutes, it was found that they have different THz absorption peaks, which basically coincide with the anomalous dispersion of the refractive index spectrum. To verify the accuracy of the experimental results, optimized stable structures and vibrational assignments were obtained through simulation calculations, and the experimental results agreed well with some of the simulation results. In addition, microfluidic chip technology was used to measure the spectral characteristics of the two sugar substitutes in solution, and a comparison was made between the solid and liquid states. It was found that the same substance exhibits correlation in different states, while the THz absorption peaks of the two substances in solution differ. Furthermore, we also compared the spectra of different concentrations and found that the THz absorption spectra of the same substance in solution are correlated with its concentration. These results fully reveal the important influence of the surrounding environment on molecular structure and its vibrational modes. This study, using mannitol and erythritol as samples, provides spectral characteristics of sugar substitutes in the low-frequency THz range and lays a foundation for the application of THz in food additive research. 

## Figures and Tables

**Table 1:** Position comparison and vibration analysis of absorption peaks.

Sample	Solid Experiment f/THz	Simulation f/THz	Vibration Analysis
Mannitol	0.94	0.95	-CH_2_OH out-of-plane bending vibration, -OH in-plane bending vibration.
	1.23	1.26	-CH_2_OH, -OH out-of-plane bending vibration
	1.73	1.62	-CH_2_OH, -OH in-plane bending vibration.
	1.96 2.17	2.03	-CH_2_OH in-plane bending vibration, -OH out-of-plane bending vibration.
	2.43	2.35	-OH in-plane bending vibration.
Erythritol	1.81	1.79	-CH_2_OH, -OH in-plane bending vibration.
	1.96	1.98	-CH_2_OH in-plane bending vibration, -OH out-of-plane bending vibration.
	2.02	2.03	-CH_2_OH, -OH in-plane bending vibration.
	2.43	2.43	-CH_2_OH, -OH out-of-plane bending vibration.

## Data Availability

The data used to support the findings of this study are available from the corresponding author upon request.
